# Analysis of Sensitive CO_2_ Pathways and Genes Related to Carbon Uptake and Accumulation in *Chlamydomonas reinhardtii* through Genomic Scale Modeling and Experimental Validation

**DOI:** 10.3389/fpls.2016.00043

**Published:** 2016-02-09

**Authors:** Flavia V. Winck, David O. Páez Melo, Diego M. Riaño-Pachón, Marina C. M. Martins, Camila Caldana, Andrés F. González Barrios

**Affiliations:** ^1^Grupo de Diseño de Productos y Procesos, Department of Chemical Engineering, Universidad de los AndesBogotá, Colombia; ^2^Group of Computational and Evolutionary Biology, Department of Biological Sciences, Universidad de los AndesBogotá, Colombia; ^3^Brazilian Bioethanol Science and Technology Laboratory, Brazilian Center for Research in Energy and MaterialsCampinas, Brazil; ^4^Max Planck Partner Group, Brazilian Bioethanol Science and Technology Laboratory, Brazilian Center for Research in Energy and MaterialsCampinas, Brazil

**Keywords:** flux balance analysis, chlamydomonas, biomass, carbon uptake, biotechnology, microalgae, bioenergy, systems biology

## Abstract

The development of microalgae sustainable applications needs better understanding of microalgae biology. Moreover, how cells coordinate their metabolism toward biomass accumulation is not fully understood. In this present study, flux balance analysis (FBA) was performed to identify sensitive metabolic pathways of *Chlamydomonas reinhardtii* under varied CO_2_ inputs. The metabolic network model of *Chlamydomonas* was updated based on the genome annotation data and sensitivity analysis revealed CO_2_ sensitive reactions. Biological experiments were performed with cells cultivated at 0.04% (air), 2.5, 5, 8, and 10% CO_2_ concentration under controlled conditions and cell growth profiles and biomass content were measured. Pigments, lipids, proteins, and starch were further quantified for the reference low (0.04%) and high (10%) CO_2_ conditions. The expression level of candidate genes of sensitive reactions was measured and validated by quantitative real time PCR. The sensitive analysis revealed mitochondrial compartment as the major affected by changes on the CO_2_ concentrations and glycolysis/gluconeogenesis, glyoxylate, and dicarboxylate metabolism among the affected metabolic pathways. Genes coding for glycerate kinase (GLYK), glycine cleavage system, H-protein (GCSH), NAD-dependent malate dehydrogenase (MDH3), low-CO_2_ inducible protein A (LCIA), carbonic anhydrase 5 (CAH5), E1 component, alpha subunit (PDC3), dual function alcohol dehydrogenase/acetaldehyde dehydrogenase (ADH1), and phosphoglucomutase (GPM2), were defined, among other genes, as sensitive nodes in the metabolic network simulations. These genes were experimentally responsive to the changes in the carbon fluxes in the system. We performed metabolomics analysis using mass spectrometry validating the modulation of carbon dioxide responsive pathways and metabolites. The changes on CO_2_ levels mostly affected the metabolism of amino acids found in the photorespiration pathway. Our updated metabolic network was compared to previous model and it showed more consistent results once considering the experimental data. Possible roles of the sensitive pathways in the biomass metabolism are discussed.

## Introduction

The increase of air emissions originated from the burning of fossil fuels and the continuous rising of the demands and prices of energy have been an issue of world impact and socio-economic importance (O'Neill and Oppenheimer, 2002; Hoegh-Guldberg and Bruno, 2010). Microalgae-based technologies focused in the bioremediation of air emissions coupled to biomass production represents a potential alternative way for reducing levels of air contaminants, creating new sources of renewable biomass that can be used for bioenergy production, or even for the accumulation of other bioproducts (Li et al., 2010; Packer et al., 2011; Scranton et al., 2015). Microalgae can, through photosynthesis, capture CO_2_ and accumulate biomass, reducing the net emission of CO_2_ during the biofuel production, contributing to protect the environment through sustainable applications, including the development of third generation biofuels (Singh and Olsen, 2011; Singh et al., 2011; Behera et al., 2014). Therefore, microalgae have attracted more attention as suitable organisms with potential to be a sustainable source of biocompounds important for a number of areas such as nutrition, aquaculture, pharmaceuticals, and biofuels. Thanks to the advances in microalgae biology, bioengineering, and molecular biology, a better understanding of metabolic routes, gene expression regulation, and cellular mechanisms is being achieved, which may contribute to further improve microalgae capabilities toward sustainable applications (Rosenberg et al., 2008).

Since the efficiency to derive biofuels from microalgae seems to be comparable to those derived from crops plants (such as soy and canola), there is a great interest to develop research in biofuel production through maximizing biomass accumulation and improving derivatization processes (Savage, 2011). Biomass can be transformed into fuels by conversion of carbohydrates to ethanol, transesterification of lipids into biodiesel, gasification of biomass to syngas, cracking of hydrocarbons, and isoprenoids to gasoline (Matsumoto et al., 2003) and the direct synthesis of hydrogen gas (Carvalho et al., 2006).

Among the many species of microalgae, *Chlamydomonas reinhardtii* has been extensively considered as a model organism to the study of different cellular mechanisms under distinct environmental conditions (Harris, 2001). This knowledge may be applied to improve specific features and could be in some cases extrapolated to close evolutionary relative species. For example, it has been found that, although *Chlamydomonas* is not considered a good lipid-storing species, under N-starvation this capability is favored (Scranton et al., 2015). Additionally, several reports have indicated that by changing the cultivation conditions, such as carbon source, light intensity, nitrogen, sulfur, and microelements availability, the biomass production could have significant improvement in microalgae cultivation (Rosenberg et al., 2008).

However, it is important to generate strategies that improve our capacity to predict the cellular behavior or to precisely identify the biological pathways that have an important role on determining biomass accumulation. Uncovering these pathways may allow us to perform permanent optimization of the capabilities of microalgae to accumulate biomass. An *in silico* method useful to analyze cell behavior under different growth conditions or disturbances and simulate metabolic changes occurring in microalgae cells consists of Flux Balance Analysis (FBA). This approach takes in consideration a linear programming strategy to solve and describe the fluxes in a systems at steady state (Orth et al., 2010).

FBA has emerged as a mathematical tool to study metabolic networks and has been successfully tested in prokaryotes including genus *Escherichia* (Edwards et al., 2002), *Lactobacillus* (Dishisha et al., 2014), *Nitrosomonas* (Perez-Garcia et al., 2014), to mention a few examples. In many of these studies, FBA was applied to detect main metabolic pathways, growth rates at specific genetic and environmental conditions, and revealed possible candidate genes for improvement of specific strains (Edwards et al., 2002). In *Chlamydomonas*, the FBA approach was previously employed to predict metabolic fluxes based on primary metabolism (Boyle and Morgan, 2009). Furthermore, FBA was used to estimate the cell growth rate at different photon flux inputs in an improved metabolic network (Chang et al., 2011).

From the perspective of the CO_2_ uptake by microalgae and biomass production, the strategies of cell survival at low CO_2_ have been well described with the characterization of the Carbon Concentrating Mechanism (CCM) (Badger et al., 1980). Through the understanding of the CCM, it is possible to explain, at least partially, the mechanisms by which microalgae are able to keep their photosynthesis performance and mitigate the stress caused by low CO_2_ supply. Basically, cell machinery elevates the CO_2_ concentration at the site of RubisCO (ribulose-bisphosphate carboxylase/oxygenase) enzyme by increasing the cellular inorganic carbon (Ci) pools favoring the carboxylase activity of RubisCO; thus, carbon fixation through photosynthesis. Moreover, some Ci transporters located in the plasma membranes and chloroplast have been suggested as main proteins on the carbon uptake process (Wang et al., 2011).

Since CCM is not fully activated at high CO_2_ concentration and considering the fact that increasing the Ci concentration inside the cell alone does not guarantee full and effective carbon fixation due to reaction saturation problems, a deeper investigation of this process through *in silico* analysis is still needed. Moreover, the knowledge about the metabolic limitations of microalgae to accumulate and fixate CO_2_ even under high CO_2_ concentrations is still incomplete. It has been previously suggested that some microalgae species are not able to fixate all carbon that is supplied to its growth and a possible saturation on the carbon fixation may occur (Melo et al., 2014). The identification of the pathways and biochemical routes that contribute to this saturation on the carbon fixation will continue to improve our understanding of the molecular control of the biomass accumulation.

Therefore, in the present study we investigated the effects of high CO_2_ concentration in a metabolic perspective. We disclosed novel routes and genes related to CO_2_ uptake and fixation through genomic scale metabolic network modeling. Our modeling strategy took in consideration the metabolic reconstruction reported by Chang et al. (2011) which was further updated and extended through homology-based sequence analysis on the annotated genome of the microalgae *C. reinhardtii*. Biomass production in this species was selected as the optimization function and evaluated under different CO_2_ concentrations, ranging from 0.04 to 10%, using experimental data of biomass content as model constrains. The results from both models were compared, sensitive genes were selected, and their relative expression experimentally validated by quantitative real-time PCR. In addition, metabolome analysis was performed for the relative quantification of primary metabolites. Together, the detection and validation of sensitive genes and pathways under high CO_2_ conditions through FBA, gene expression analysis and metabolomics, indicated that CCM, photorespiration and mitochondrial related processes have important roles in the control of biomass accumulation in *C. reinhardtii*. Our work revealed potential candidate pathways and genes for future maximization of microalgae biomass production.

## Materials and methods

### Metabolic network reconstruction

The *i*RC1080 model from Chang et al. (2011) was supplemented by genomic information and gap filling using metabolic databases. All the annotated protein sequences for *C. reinhardtii* (14,414 sequences) were retrieved from NCBI database (Sayers et al., 2009) and served as input for previous scripts developed in our research group that identify enzymes based on homology analysis by using BLASTp from NCBI. This analysis identified a total of 1632 enzymes (Supplemental Table 1). The recognized proteins were then used to extract all the metabolic reactions associated according to the KEGG database (Kanehisa and Goto, 2000) (Supplemental Table 2). An improved manual curation was performed due to the appearance of new reactions, substrates, and products that required new connections. Manual inspection was also needed to avoid generic metabolites and repeated reactions. The directionality of the new reactions was defined by the criteria of the free Gibb's energy at 27°C and pH 7 and calculated by the group contribution method as described in Mavrovouniotis (1990). Compartmentalization was needed to guarantee the placement and viability of the reaction according to Chang et al. (2011), literature and databases based on signal peptide (Petersen et al., 2011). Exchange reactions were included in order to connect the new metabolites in the cellular compartments ensuring origin and consumption of reagents. Special attention was required for lipids and carbohydrates because generic or general names were found in KEGG database hindering the integration of new reactions.

### Sensitivity analysis

The two metabolic networks, the *i*RC1080 model given by Chang et al. (2011) and our complemented model, were reconstructed into the stoichiometric matrix and the fluxes of the transport reactions adjusted to represent autotrophic growth conditions. Light condition was fixed at cool-white fluorescent (57.54 mE/gDW.h, equivalent to 400 μE·m^−2^·s^−1^) and the CO_2_ fluxes modified to evaluate the effects of different conditions of CO_2_ supply at the steady state of the cell metabolism and other parameters were established as previously described (Melo et al., 2014). In both cases, the Biomass function was not modified and was implemented as proposed by Chang et al. (2011). The lower and upper bounds of the new reactions were fixed according to standard values. The optimization problem was resolved using Xpress IVE® by setting constraints as follows:

$$\begin{array}{c} \max_{\text{z}}c^{T}v\left(1\right)\\ subject\;to\;Sv=0\\ LB\leq v\leq UB\\ c^{T}\in R^{n}|c^{T}=\left[0\;0\;0\ldots 1\ldots 0\;0\;0\right]|\;pos\left(1\right)=Biomass\;reaction\\ v\in R^{n}\\ S\in R^{m\times n}\\ LB\in R^{n} \end{array}$$

Where ***S*** is the stoichiometric matrix, ***v*** is the flux vector, *LB*: lower bound, *UB*: Upper bound, *c* is a vector of zeros that sets the objective function, **m** is the number of metabolites, **n** is the number of reactions, pos(1) is the objective function (biomass).

The definition of candidate metabolic pathways and genes that could play an important role in CO_2_ level response was performed through the identification of reactions affected under different CO_2_ fluxes through FBA simulations. FBA was resolved at five different CO_2_ conditions (0, 2.5, 8, 5, and 10% CO_2_) and the resulting magnitudes defined the CO_2_-sensitive reactions by the flux variation coefficient ρ [set to be significant when ρ ≥ 0.01 (Melo et al., 2014)]. Experimental data of total biomass content were considered as model constrains for calculating the CO_2_ fluxes, as previously described (Melo et al., 2014). The results from both models were compared to analyze the effects of the metabolic networks on the sensitivity of FBA.

Results from metabolomics analysis were used to further verify our sensitive reactions and support the FBA analysis. Thus, metabolites relatively quantified by metabolomics were compared to check compatibility between overexpression and knock-down according to our FBA results. For this, all the FBA-sensitive reactions for one specific metabolite were grouped with their calculated fluxes at low and high CO_2_ concentrations. A net flux was then calculated by adding production reactions, subtracting consumer reactions, and a rate between the net flux at low CO_2_ over the net flux at high CO_2_ was finally considered.

### Cell strain and culture conditions

The *C. reinhardtii* strain CC503 cw92mt+ (Chlamydomonas Resource Center University of Minnesota, USA) was cultivated in HSM medium at 27°C in autotrophic growth conditions, under different CO_2_ concentrations [0.04 (air), 2.5, 5, 8, and 10% CO_2_] in a R'ALF Plus solo 6.7 L bioreactor (Bioengineering, Inc., USA) in constant and continuous illumination with cool-white LED (average 400 μE·m^−2^·s^−1^). The cultures remained in a batch mode with starting culture volume of 4 L, in open system with automatic controlled CO_2_ gas flow in air, no pH control and continuous stir at 60 rpm. Cell inoculation was carried out with a 10 mL pre-inoculum taken from a sample at steady state. Cells were harvested by centrifugation at the late exponential phase of cell growth (O.D._750nm_ ≈ 0.9) for biomass characterization.

Cell growth monitoring was performed daily with measurements of O.D. at 750 nm and cell counting using Neubauer chamber. Growth rates were calculated as previously described (Sorokin and Krauss, 1958).

### Total biomass measurement

Cell pellets from 50-mL cell culture aliquots at the late exponential phase were washed three times with 2 mL of de-mineralized water to remove inorganic salts and the final cell suspension was transferred to previously weigh empty Petri dishes. Plates containing the biomass were kept overnight at 90°C, and the dry weight was measured. Three (0.04 and 10% CO_2_ conditions) or two biological replicates (2.5, 5, and 8% CO_2_ conditions), each one with three technical replicates, were considered for statistical analysis.

### Protein, lipid, pigment, and starch measurements

Cells at low (0.04%) and high CO_2_ (10%) were harvested in 50-mL aliquots (three biological replicates and three technical replicates per sample) and centrifuged at 3000 × g in a swing rotor bench centrifuge for 5 min at 4°C. Cell pellets were kept at −80°C until further processing. Frozen cell pellets were macerated until conversion into a light green powder. Protein extraction was performed using a buffer containing 100 mM Tris pH 7.5, 4 mM EDTA, 5 mM 2-mercaptoethanol, 10% glycerol, and 0.05% Triton X-100. Protein content was determined by Bradford assay (Bradford, 1976). Pigments were extracted using ethanol and quantified using Kaczmar equations (Henriques et al., 2007). Lipids and carbohydrates were extracted using chloroform:methanol:water (1:2:0.8) mixture, and the resulting chloroform layer, which contains the lipids, was evaporated in a vacuum oven at 30°C for 24 h. The total lipid content was calculated by dry weight. Starch content was determined in the insoluble fraction after the chloroform:methanol:water extraction and solubilized in 0.1 M NaOH for 30 min at 95°C. After neutralization, starch was digested to glucose by the addition of amyloglucosidase and α-amylase at 37°C overnight. Determination of the glucose released by the enzymatic digestion of starch was assayed enzymatically by coupling to reduction of NADP+ to NADPH in a microplate reader (Stitt et al., 1989).

### Gene expression analysis by quantitative RT-PCR

Based on our results on the sensitivity analysis using FBA approach on the model described by Chang et al. (2011) and literature review, 40 sensitive genes related to CO_2_ changes [low CO_2_ (0.04%; air) and high CO_2_ (10% CO_2_)] were selected to further gene expression analysis through the relative quantification by qRT-PCR. These candidate genes were previously annotated to the following routes and mechanisms: CCM, Calvin cycle, Glycolysis/Gluconeogenesis metabolism, and Glyoxylate/dicarboxylated.

Cells for RNA extraction were harvested in 2-mL aliquots by centrifugation for 2 min, 3000 × g, at 4°C, and the cell pellets were immediately frozen and kept at −80°C until further use. Total RNA extraction was performed using the RNeasy Plant Mini Kit (Qiagen, Hilden, Germany) as previously described (Winck et al., 2013). Total RNA samples were treated with TURBO DNAse (Ambion, Darmstadt, Germany) as indicated by the manufacturer. Absence of genomic DNA contamination was accessed by qRT-PCR using primers annealing to an intergenic region of chromosome 16 (IGR1, IGR2). Primers were designed using QuantPrime tool (www.quantprime.de) following the criteria as follows: Tm = 60 ± 1°C, length 18–25 bases, preferentially on exon-exon junctions. When possible, primers were designed to have a GC content of 45–55%, generating a single PCR product sizing between 60 and 150 bp. Primers were synthesized by Macrogen (Macrogen, Korea).

Three micrograms of total RNA were used for cDNA synthesis employing the SuperScript III First Strand System (Invitrogen, Darmstadt, Germany) according to the manufacturer's instructions, using oligo-(dT_20_) as primer for the synthesis of the first complementary DNA strand. Two genes [Actin (ACT) and Ubiquitin protein ligase (UBQ)] were selected as reference genes. All the cDNA samples were amplified in 96-well plates in an Applied BioSystems ABI7500 FAST system. The qPCR reaction was carried out in 10 μL containing 1 μM primers and SYBR Green qPCR Master Mix (Roche). The primer sequences used in this study are presented in Supplemental Table 3. Real-time PCR reaction parameter settings were as follow: 2 min at 50°C, 10 min at 95°C, followed by 40 cycles of 15 s at 95°C and 1 min at 60°C. Amplicons which dissociation curve resulted in double melting temperatures or doubled products in a 4% gel electrophoresis were discarded from further analysis. The relative expression ratio for each gene was calculated as previously described (Pfaffl, 2001). The PCR efficiency for each reaction was calculated based on the profile of the emitted fluorescence in the exponential phase (Rutledge and Stewart, 2008; Rutledge, 2011). Three biological replicates, each one with one technical replicate for the two conditions analyzed (0.04 and 10% CO_2_) were performed.

### Metabolomics analysis

Aliquots of 50-mg pellet from cell culture were taken in the stationary phase (OD_750_ ≈ 0.9) at low (0.04%) and high CO_2_ concentration (10%). Metabolites were extracted with methanol-chloroform-water HPLC grade (2.5:1:1.4) mixture and three biological replicates for low CO_2_ (0.04%) and two biological replicates for high CO_2_ (10%) were considered. Samples were immediately frozen in liquid nitrogen and lyophilized for storage. Extraction and derivatization of metabolites were performed as outlined previously (Lisec et al., 2006). GC-TOF-MS data were obtained using a PAL-Combi XT autosampler (PAL System http://www.palsystem.com/), coupled to an Agilent 7890 A gas chromatograph—Leco Pegasus HT time-of-flight mass spectrometer (LECO, St. Joseph, MI, USA; http://www.leco.com/). Identical chromatogram acquisition parameters were used as those previously described (Weckwerth et al., 2004). Chromatograms were exported from Leco ChromaTOF software (version 4.51.6.0) to R software. Peak detection, retention time alignment, and library matching were obtained using the TargetSearch package from bioconductor (Cuadros-Inostroza et al., 2009). Data obtained from GC-TOF-MS analysis were normalized by cell number, followed by sample total ion content (TIC) as described previously (Giavalisco et al., 2011).

## Results

### Metabolic reconstruction revealed novel reactions

Inspections through different metabolic databases [GoFORSYS—ChlamyCyc (http://chlamycyc.mpimp-golm.mpg.de/) or PMN (http://pmn.plantcyc.org/CHLAMY/) that report proteins and curated metabolic data, showed that only a small group of enzymes of *Chlamydomonas* has been discovered and functionally characterized. Considering this, our homology analysis was performed comparing all annotated proteins in KEGG database to identify the EC numbers and the associated reactions. In order to simplify the reconstruction, only the reactions with reported reactants in Chang et al. (2011) were considered.

**Figure 1:**
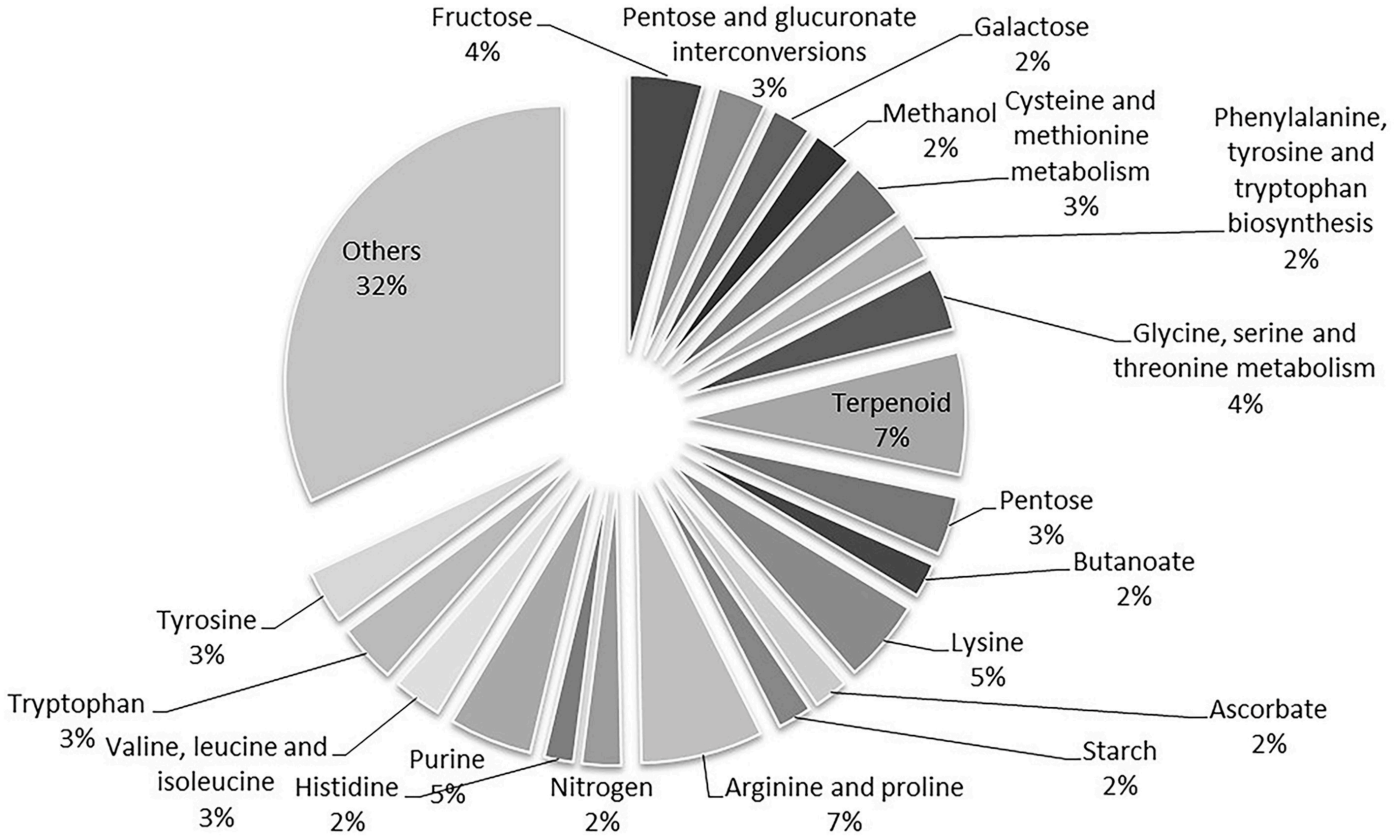
**Percentage contribution of the additional reactions included in the complemented metabolic network based on annotation of genes and substrates**. The whole annotated genome was considered to identify enzymes by homology analysis. Only reactions with identified reactants in Chang et al. (2011) were considered.

A total of 1632 enzymes were found by BLASTp analysis using our script and a total of 2599 reactions (without compartmentalization) were associated. After removing the reactions that already exist in the work of Chang et al. (2011), 1803 new reactions were identified. By following the proposed criteria, considering only the reactions with identified reactants described by Chang et al. (2011), we detected 1380 new compartmentalized reactions based on the homology analysis with KEGG database (Supplemental Table 2). Some products had to be connected by gap filling with manual inspection in KEGG database taking the shortest pathway for FBA purposes. The reactions were compartmentalized ensuring availability of substrate within the 10 compartments and a total of 87 reactions were defined as reversible based on Gibbs energy evaluation and previously reported studies. Figure 1 depicts the percentage contribution of the main reaction categories, including substrates, and products for the updated of the metabolic network.

We found that some reactions and metabolites have generic names in KEGG database, especially those associated to lipid and starch metabolism. This inconsistent information provides new reactions and metabolites that cannot be interconnected affecting viable results from the FBA analysis. Gaps within the different pathways were observed when looking at the rows of the stoichiometric matrix and manual interconnection had to be assumed to consume products based on metabolic maps. Furthermore, unlikely reactions were found associated to homology parameters, reactions belonging to penicillin, and cephalosporin biosynthesis were rejected because no information was confronted with *Chlamydomonas*. These results indicated that manual curation is still necessary and mandatory to guarantee feasible models.

### Curation level of the network affects FBA sensitivity analysis

**Figure 2:**
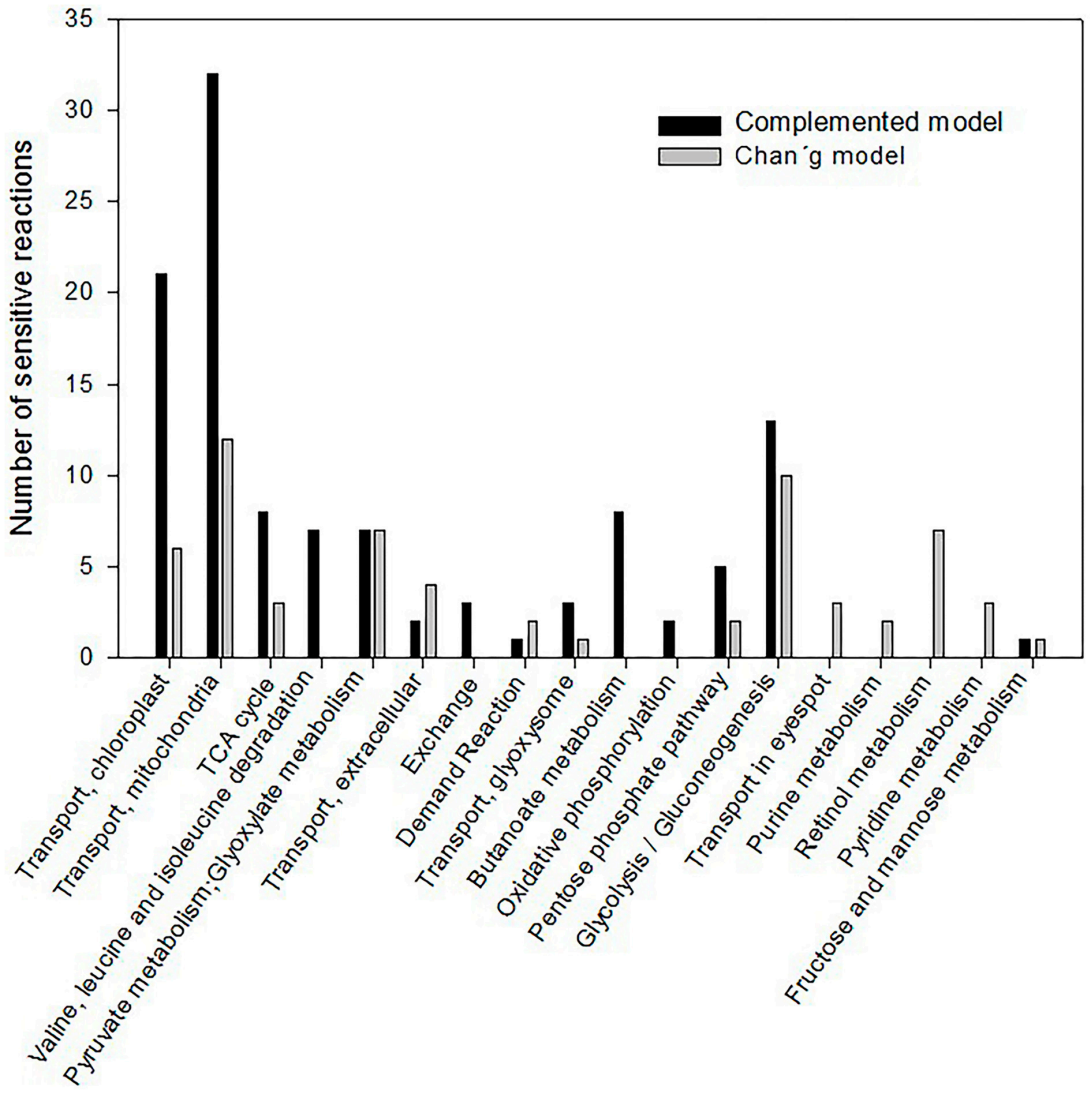
**Distribution of the metabolic pathways affected on sensitive analysis from the both metabolic models evaluated**. FBA analysis was performed for the different CO_2_ inputs in the system at autotrophic conditions. The same biomass function was established in both models and sensitive reactions were identified based on the flux variation coefficient. Metabolic pathways affected on sensitive analysis were annotated and the number of sensitive reactions is indicated.

In a previous work, we have identified a total of 87 sensitive reactions solving the biomass function (Melo et al., 2014). A comparison of these sensitive reactions was performed against the previous model proposed by Chang et al. (2011), and 155 sensitive reactions were identified in our current complemented network (Supplemental Table 4). The percentage distribution of the annotation of the metabolic pathways corresponding to the sensitive reactions is shown (Figure 2). Data tendency is highly conserved between both models: reactions associated to mitochondrial transport, chloroplast transport, glycolysis/gluconeogenesis, and TCA cycle are both subjected to regulation under different CO_2_ input fluxes. However, in our complemented reconstruction, more, and novel sensitive reactions appeared related to the amino acid metabolism of alanine, threonine, tryptophan, and propionate metabolism (resulting in production of valine and alanine) (Supplemental Table 5). Other reactions belonging to starch, sucrose, and methane metabolism were also glimpsed. Over 25 genes remained sensitive in both reconstructions and new ones were identified (Table 1). Our results showed that larger networks may lead to different results on the sensitive analysis and may contribute to disclosing new candidate sensitive reactions.

**Table 1:** **Sensitive genes from FBA analysis of the complemented network**.

**Biological processes**	Sensitive genes^*^
Transport, mitochondria	**MITC14**/MITC18/PTB8/PTB7/PTB1/PTB12/PTB4/PTB2/CRv4_Au5.213.g4507.t1
Phenylalanine tyrosine and tryptophan	**AST4/HIS5**
TCA cycle/CO_2_ fixation	**ACH1**/IDH3/SDH1/SDH2/OGD1
Valine, leucine, and isoleucine degradation	CRv4_Au5.s4.g11844.t1/Crv4_Au5.s12.g3863.t1/CRv4_Au5.s6.g13618.t1/CRv4_Au5.s12.g3863.t1/g1910.t1
Pyruvate metabolism; Glyoxylate metabolism	**HYDA1/MFDX/HYDA2**/PFL1/ACK2/AACK1/ACK1/PAT1/PAT2/CRv4_Au5.s6.g13230.t1/CRv4_Au5.s2.g9723.t1
Alanine and aspartate metabolism; glycerine, serine, and threonine	**AST3/AST1**
Carbon fixation	AAT1/**AAT2**/MME3/MME6/MDH5/MME2
Glycolisis, Gluconeogenesis, Valine, Leucine, and isoleucine degradation	**DLDH1**/PDC2/PDH2/ALSS1/ALSL1/PYK1/PYK5/PHG1/GAP3/GAP1/PGM2/PGM5/PGM1B/PGK1/TPIC/FBA1/FBA2/PGI1/GPM2
Transport, extracellular	**NAR1.6/NAR1.3/NAR1.4**
Pentose phosphate pathway	TAL1/TRK1/RPE1/RPI1
Glycine, serine, and threonine metabolism	Crv4_Au5.s10.g124.t2/THD1/SHMT3
Transport, chloroplast	AOC6/**AOC5/AOT7/DAT1**/OMT1/AOT5/**FBB13/**NAR1.5/NAR1.2/NAR1.1/AAA3/AAA1/CRv4_Au5.s14.g5515.t1/CRv4_Au5.s15.g5921.t1/CRv4_Au5.g14736.t1/MOT20/**MIP1/MIP2**
Butanoate metabolism	CRv4_Au5.s7.g14479.t1/CRv4_Au5.s16.g6952.t1
Oxidative phosphorylation	**NDA3/NUO11/NUO10/NUO13/NUO21/NUO3/NUO5/**NUO6/NUO8/NUO9/IPY1/IPY3
Propanoate metabolism	PFL1
Nitrogen metabolism	CGL77/IBA57/GCST

* *Bold type names represent common sensitive genes present in both metabolic reconstructions of Chang et al. (2011) and our present complemented reconstructed network*.

Our sensitive analysis revealed that mitochondria is the most sensitive cellular compartment and showed the highest number of reactions affected by varying CO_2_ concentrations, mainly related to amino acid transporters, carriers (phosphate, dicarboxylate), and transport of compounds such as ethanol, ammonia, and O_2_. The mitochondria is important to the maintenance of intracellular redox gradients, impacting the rates of photorespiration, and efficiency of photosynthesis (Araújo et al., 2014). Therefore, our results of the sensitive analysis pointed to a possible role of mitochondria in modulating the biomass production in microalgae. In order to validate our results from the FBA analysis, the biomass objective function was compared with the experimental data on biomass production. As we have previously shown (Melo et al., 2014), the magnitudes of the growth rates in our simulations were similar to experimental values. However, *in silico* data showed a linear increment of biomass production that does not represent the saturation trends observed in our experimental conditions. High CO_2_ concentration enhances biomass production through non-linear trend.

**Figure 3:**
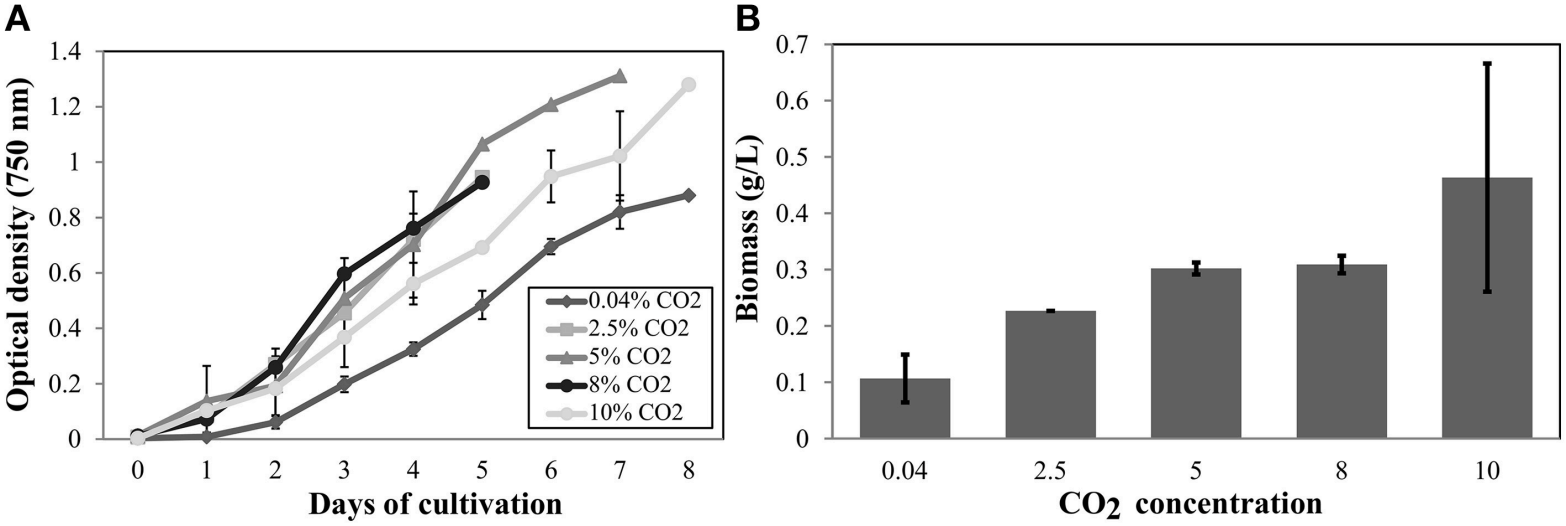
**Cell growth and biomass profiles at different CO_2_ inputs in autotrophic conditions**. **(A)** Cells were grown in a controlled bioreactor and autotrophic conditions (in HSM medium). Absorbance at 750 nm was daily measured. **(B)** Dry weight biomass (gDW/L). The data show the average of two biological replicates and three technical replicas for each sample. Error bars indicate standard deviation.

**Figure 4:**
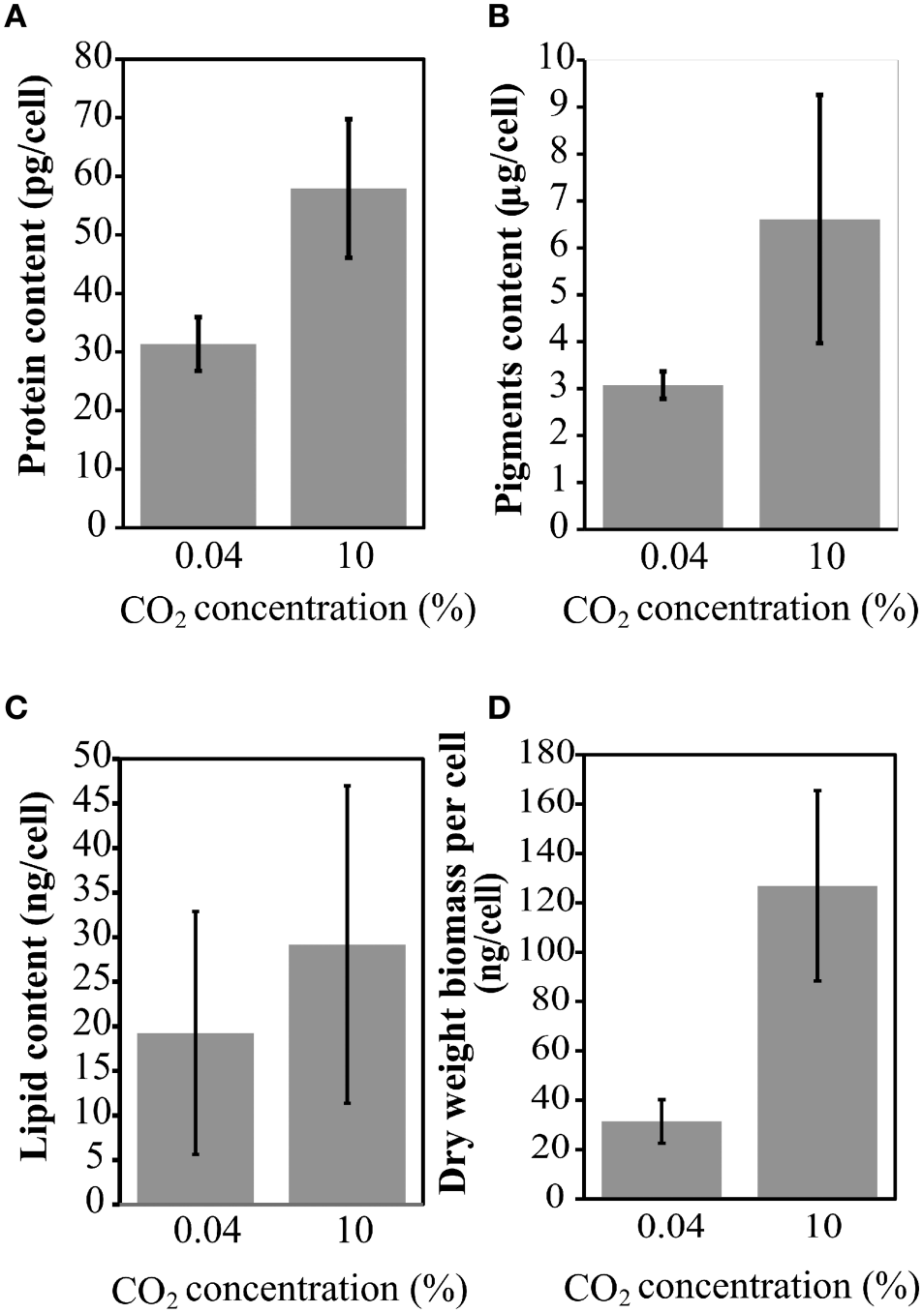
**Protein, lipid, and pigment content at 0.04 and 10% CO_2_ concentrations**. **(A)** Protein content; **(B)** Total pigments; **(C)** Total lipid content; **(D)** Dry weight biomass per cell. Three biological replicates with three technical replicates were computed. Error bars indicate standard deviation.

Cell growth was measured daily (Figure 3A) and a significant increase in the total dry weight biomass was evident at high CO_2_ concentrations (above 0.04% CO_2_), reaching the highest content when evaluated at 10% (Figure 3B). Therefore, there was a biomass increment of at least 300% in the cell culture in high CO_2_ concentrations compared to cells cultured under low CO_2_ concentration (air); this information corroborate previous results (Chang et al., 2011). The amount of proteins (Figure 4A), pigments (Figure 4B), lipids (Figure 4C), and dry weight (DW) (Figure 4D), quantified per cell and compared between the low CO_2_ (0.04%) and high (10%) CO_2_ conditions, showed a positive increase at high CO_2_ concentration. However, no significant changes were found for starch (about 17 μmol/g or mg/dDW ± 2.5 at low CO_2_ and 16 μmol/dDW ± 4.0 at high CO_2_, *p* > 0.05). Moreover, we observed that cell culture at 5% CO_2_ produced similar amounts of total biomass than cells cultured at 10% CO_2_, suggesting the existence of a saturation trend in the biomass production once cells are cultivated in CO_2_ concentration higher than 5%.

Furthermore, the cell growth curves observed on high CO_2_ conditions (2.5, 5, 8, and 10% CO_2_) showed similar profiles but cellular density was higher and statistically different from those cell cultures under low CO_2_ (0.04%). Thus, the exposure to high CO_2_ lead to an increased number of cells per culture volume and enhanced cellular capability to accumulate biomass, likely affecting mitosis-related cell cycle and energy metabolism.

### Gene expression analysis revealed candidate sensitive genes

The experimental validation of our results on the identification of CO_2_ sensitive metabolic reactions included gene expression analysis of selected genes and a metabolomics approach focused in the identification and relative quantification of the main metabolites of primary metabolism.

**Figure 5:**
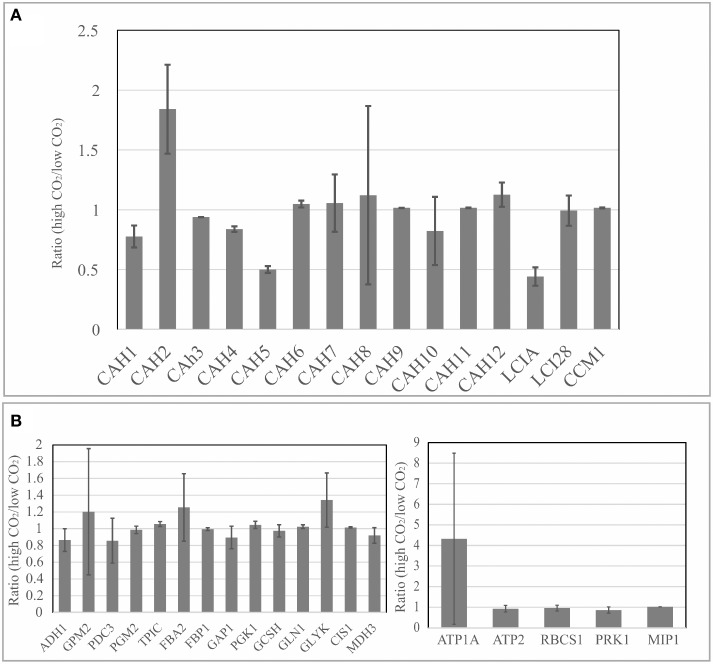
**Gene expression analysis through real-time qPCR**. **(A)** Relative expression levels of genes related to carbon concentrating mechanism were compared at low (0.04%) and high CO_2_ concentrations (10%); **(B)** Expression levels of genes related to glycolysis/gluconeogenesis and Calvin cycle were compared between low (0.04%) and high CO_2_ concentrations (10%). Data normalization was performed using the expression level of gene coding for Actin (housekeeping gene) as reference for relative gene expression calculations. Three biological replicates were analyzed with two technical replicates. Error bars indicate standard deviation.

In Figure 5A, the relative expression levels of genes encoding carbonic anhydrases (CAHs) are shown. Carbonic anhydrases can catalyze the reversible interconversion of carbon dioxide to carbonic acid in order to increase the carbon uptake and availability in the site of photosynthesis at the chloroplast. The analysis of these genes was included because there were previous evidences that CO_2_ related mechanisms may be determinant to biomass accumulation and CAHs play an important role on cellular carbon uptake (Fang et al., 2012; Winck et al., 2013). Our results showed that gene transcripts for CAH1, CAH4, CAH5, and LCI1 were found to be more abundant in low CO_2_ concentration (0.04%). Similar results were shown by previous studies on the Carbon Concentrating Mechanism (Brueggeman et al., 2012). Moreover, our updated FBA sensitivity analysis revealed that metabolic pathways of glycolysis/gluconeogenesis, glyoxylate, and dicarboxylate metabolism are affected by the changes in CO_2_ concentration. Genes coding for glycerate kinase (GLYK), glycine cleavage system, H-protein (GCSH), NAD-dependent malate dehydrogenase (MDH3), low-CO_2_ inducible protein A (LCIA), low-CO_2_-inducible protein 23 (LCI23), mitochondrial pyruvate dehydrogenase complex, E1 component, alpha subunit (PDC3), fructose-1,6-bisphosphate aldolase (FBA2), glyceraldehyde-3-phosphate dehydrogenase (GAP1), dual function alcohol dehydrogenase/acetaldehyde dehydrogenase (ADH1), and phosphoglucomutase (GPM2), were defined as sensitive nodes in the metabolic network simulations and found to be responsive to the changes in the carbon fluxes in the system.

As discussed previously, the sensitivity analysis may generate different outputs depending on the completeness of the metabolic network used as input for simulation. Genes coding for GLN1, CIS1, ATP2, PRK1, and FBP1, which were found to be sensitive in Chang et al. (2011), showed low variation in the gene expression levels in our network model (Figure 5B). These genes are involved in different metabolic processes such as carbon fixation, glutamate metabolism, and fructose metabolism, as previously shown (Melo et al., 2014). Moreover, genes coding for glycerate kinase (GLYK), phosphoglucomutase (GPM2), fructose-1,6-bisphosphate aldolase (FBA2), and glyceraldehyde-3-phosphate dehydrogenase (GAP1), sensitive in both metabolic network models compared, showed variations at the gene expression levels. This may suggest that our proposed network model could give complementary and valuable insights considering the results of transcriptional changes.

### Metabolites are affected by changes on CO_2_ concentration levels

The identification of sensitive reactions through FBA guided us to perform a quantitative analysis of the metabolites of cells cultured in low (0.04%) and high (10%) CO_2_ concentrations. The metabolomics analysis performed using GC-TOF-MS permitted the quantification of 67 metabolites in the two compared CO_2_ concentrations (Supplemental Table 6).

We further explored the CO_2_ sensitive reactions of 13 out of the 67 metabolites identified. Our model was able to predict the behavior of these 13 metabolites at high and low CO_2_ concentrations regarding the amount and presence within the cells, being consistent with experimental results at the two growth conditions, except for the measurements of glycine, isocitrate, and sucrose, which were not good represented by the FBA.

Other 50 metabolites quantified did not show significant differences between the two growth conditions (0.04 and 10% CO_2_) and 50% of these did not show any alteration through FBA. From this perspective, it is shown that the model assumptions in general terms seem to be consistent with real behavior of maximizing biomass. However, others mechanisms may be activated resulting in saturation trends for CO_2_ processing and constraints.

**Figure 6:**
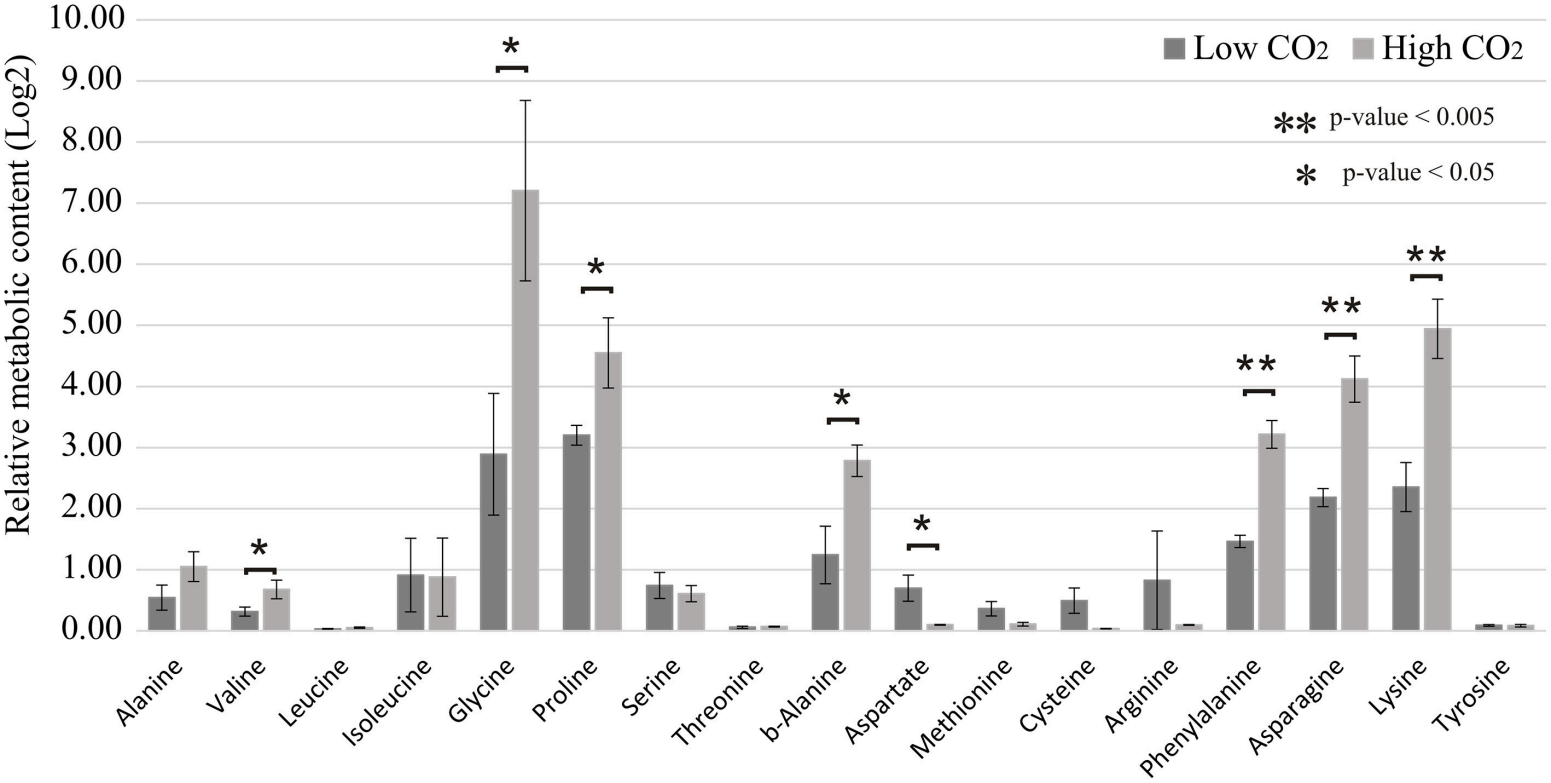
**Relative quantification of amino acids in low and high CO_2_ concentrations**. Metabolomics analysis was performed for the cells cultivated under low (0.04%) and high (10%) CO_2_ concentrations. Amino acid content was measured by mass spectrometry analysis. Data is presented in Log_2_ scale. Three biological replicates for low CO_2_ (0.04%) and two biological replicates for high CO_2_ (10%) were considered and three technical replicates were considered for each sample. Error bars indicate standard error.

**Figure 7:**
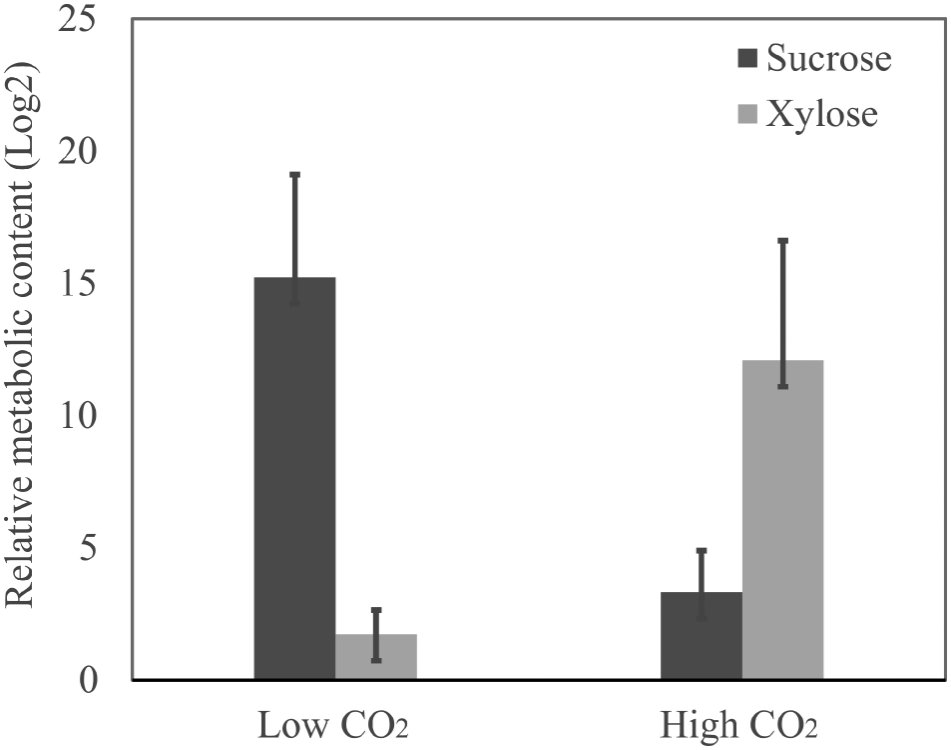
**Sucrose and xylose relative content in cells at low and high CO_2_ concentrations**. The relative content of sucrose and xylose was determined by metabolomics analysis through mass spectrometry. Data is presented in Log_2_ scale. Three biological replicates for low CO_2_ (0.04%) and two biological replicates for high CO_2_ (10%) were considered and three technical replicates were considered for each sample. Error bars indicate standard error.

We observed that the amino acids glycine, proline, b-alanine, phenylalanine, asparagine, and lysine are the ones that suffered the most prominent alterations in abundance in response to high CO_2_ concentration (Figure 6) (Supplemental Table 7). Relative levels of sucrose have been significantly reduced and the amount of xylose increased in the cells cultivated at 10% CO_2_ concentration. This is an important indication of the cellular metabolic shift at low and high CO_2_ concentrations. We also observed that glycerate was more abundant in cells at 10% CO_2._ Moreover, xylose, a potential inhibitor of photosynthesis, was more than five-fold more abundant in cells at 10% CO_2_ (Figure 7). These results suggest that photorespiration or an alternative pathway with similar substrates and products may be modulated in cells at high CO_2_ concentration, possibly leading to the saturation trend in the biomass accumulation observed in cells under this condition.

FBA analysis indicated that at low and high CO_2_ concentrations the metabolic routes related to photorespiration may remain at least partially activated (it was not completely off) in order to satisfy the model constraints. The magnitude of the fluxes are >0 and are contributing to maximize the biomass function. Special attention to those magnitudes were taken into account for glycine transport from chloroplast to mitochondria, serine transport from mitochondria to chloroplast, hydroxypyruvate production, glycerate, and 3-phospoglycerate production in the chloroplast. All these processes may be related to photorespiration and showed positive fluxes in both Chang et al. and our complemented metabolic network models. Although genomic-scale restriction was not considered in the FBA, it is shown that these routes contribute to maximize biomass production.

## Discussion

In the present work, we compared the effects of varying CO_2_ concentrations in the biomass composition of *C. reinhardtii* cells. Moreover, we identified candidate genes sensitive to the variations on the CO_2_ concentrations through the use of FBA in an extended metabolic network model presented here. Further experimental validation through gene expression analysis and metabolomics was performed. Our experimental results suggested that cells at high CO_2_ have increased capability toward biomass production. However, it also indicated that cells cultivated at CO_2_ concentrations higher than 5% achieved a saturation trend on total biomass accumulation. Although our FBA model was unable to describe this saturation trend, the magnitudes of the growth rate values were consistent between the different CO_2_ concentrations compared. This may be explained by the fact that the model was resolved using linear optimization and a change in input values, such as any external metabolite, represents a proportional increase in the objective function.

Our results on the FBA sensitivity analysis of the two metabolic reconstructions showed a significant increase of flux in metabolic routes occurring in the chloroplast and mitochondria transport systems, including TCA cycle, glycolysis/gluconeogenesis, and amino acids biosynthesis in cells under high CO_2_ concentrations. For the new sensitive routes and genes identified by our FBA, it was noted that many reactions were associated with energy metabolism. These sensitive routes have a number of genes which expression may be affected. Therefore, these genes may be interesting candidates on further biotechnological applications focused in the enhancement of biomass production. Reactions associated to the transport of amino acids, pyruvate, and carboxylate species into the mitochondria and chloroplast were shown to be the most sensitive ones. Moreover, mitochondria resulted as the most sensitive compartment as most reactions detected in our sensitive analysis may occur inside this organelle. Mitochondria presents a fundamental role in growth and biomass production, through its role on energy metabolism.

Sensitivity analysis also revealed a high dependence on the metabolic network quality and completeness on the identification of key routes. These routes can vary in differing models making it difficult to achieve consistent results; however, consensus in some main nodes were found and validated by qRT-PCR. Thus, our results revealed novel biochemical routes and candidate genes that may be relate to biomass production, through the modulation of the rate of biosynthetic processes.

The changes observed in the metabolite profiles of cells at low (0.04%) and high CO_2_ (10%) concentrations suggest that high CO_2_ concentration in microalgae cell culture may trigger mechanisms that are able to control the carbon fixation by the alternative synthesis of compounds that may have an inhibitory effect on the photosynthesis, or may enhance energy losses through photorespiration. These processes may have a role in the control of the cell biomass content and microalgae cell population, even under non-limiting availability of CO_2_. Further experiments are now necessary to provide more evidences of these effects.

Experimental validation also confirmed variations in the gene expression profiles of selected genes when cells are cultivated at different CO_2_ concentrations. It was observed that many CCM-related genes are overexpressed at low CO_2_, indicating, as expected, that cells change their metabolism to produce enzymes involved in enhanced carbon uptake and carbonic acid conversion, instead of redirecting energy to build biomass precursors. A previous transcriptomics analysis compared *Chlamydomonas* cells at high (5%), low (0.05%), and very low (0.02%) CO_2_ concentrations (Fang et al., 2012) and revealed that the wild type strain cc125 vs. a cia5 mutant strain cc2702 showed at least 345 genes differentially expressed from low vs. high CO_2_ and 696 genes from very low vs. high CO_2_ in wild type cells. Several of those genes were found in our list of sensitive genes as it is summarized in Table 2.

**Table 2:** Candidate CO_2_ sensitive genes which were identified as differentially expressed in a transcriptome dataset previously published comparing cells at high vs. low CO_2_ concentrations using RNA-seq^*^.

**Metabolic pathway or biological process Description**	**Candidate CO_2_ sensitive genes**	
	**Complemented network (present work)**	**Chang et al., 2011**
Mitochondrial transport	MIT28	
	PTB12	
	PTB4	
	PTB2	
Phenylalanine, tyrosine, and tryptophan biosynthesis	AST4	
Carbon fixation	MDH5	RBCS1
Pentose phosphate pathway	TAL1	*RPE1*
	*RPE1*	
	RPI1	
Transport, chloroplast	DAT1	
	NAR1.2	
Oxidative phosphorylation	NDA3	
	IPY1	
	IPY3	
Glycolysis, gluconeogenesis, valine, leucine, and isoleucine degradation	*PGK1*	*PGK1*
Extracellular transport		PTA3
		PTA4
Glycine, serine, and threonine metabolism		GCSP
		THS1
Glyoxylate metabolism		GLYK
Prphyrin and chlorophyll metabolism		GSA

* *Transcriptome dataset previously published (Fang et al., 2012)*.

Our results on the gene expression of carbonic anhydrases and genes related to CCM were consistent with previous findings. It was confirmed that the expression of CCM1 (or CIA5) itself does not depend on the CO_2_ level (Fang et al., 2012). Previous studies have shown that proteins CAH1, CAH3, CAH4, CAH5, and CAH6 are responsive to variations on the CO_2_ concentration. Moreover, protein LCIA, reported as induced at low CO_2_, encodes a format/nitrite transporter that increase HCO3- transport in the stroma (CIA) was found highly expressed at low CO_2_ concentration (Fang et al., 2012).

Besides the identification and validation of the expression of the main CA's under low and high CO_2_ conditions, we further compared which metabolites were modulated in the two CO_2_ concentrations (0.04 and 10%). Our metabolomics analysis indicated that the concentration of metabolites possibly related to photorespiration or other alternative route is modulated in response to high CO_2_ concentration.

Our results on the experimental biomass characterization showed that the pigment content per cell is the most sensitive component and its amount is almost duplicated at high CO_2_ concentration, indicating possible enhanced needs for light absorption and carbon fixation. However, starch content showed no significant changes which imply that the continuous light conditions may lead cells to control their carbohydrate stocks, probably due to the absence of a dark period and reduced need for starch accumulation or through the activation of alternative processes of carbon usage.

Altogether, these findings suggest that biomass accumulation does not enhances indefinitely with the enhanced availability of CO_2_. The control of biomass accumulation may be closely connected to the regulation of biochemical pathways occurring in the mitochondria and the use of energy sources toward the accumulation of proteins and pigments.

## Author contributions

FW, AG: conceived and designed the work; FW, DP, MM: performed experiments; FW and DP: drafted the manuscript; CC: performed metabolomics analysis; FW, DR, DP, AG: performed data analysis. All authors have read and approved the final version of the manuscript.

## Funding

This project was funded by Universidad de los Andes and FAPESP.

### Conflict of interest statement

The authors declare that the research was conducted in the absence of any commercial or financial relationships that could be construed as a potential conflict of interest.
